# A Case of Chemical Pregnancy in a Female With Turner Syndrome

**DOI:** 10.7759/cureus.47172

**Published:** 2023-10-17

**Authors:** Sravya Gudapati, Kamlesh Chaudhari, Apoorva Dave, Shazia Mohammad, Shaikh Muneeba

**Affiliations:** 1 Department of Obstetrics and Gynaecology, Jawaharlal Nehru Medical College, Datta Meghe Institute of Higher Education and Research, Wardha, IND

**Keywords:** turner syndrome, primary amenorrhea, streak gonads, chromosomal abnormality, primary infertility

## Abstract

Turner syndrome (TS) is a genetic anomaly that is characterized by the absence of an X chromosome, either completely or partially. Primary amenorrhea, short stature, webbed neck, cubitus valgus, and a little intellectual disability are some of the characteristics. Infertility is also one of the most common clinical symptoms of TS-affected females. With the advent of assisted reproductive technology (ART), chances of childbearing possibilities for TS females have risen. Infertility issues in females with TS are challenging, but they can be managed with proper counseling and ART by artificial implantation, oocyte donation, and others. This case report aims to present the case of a 27-year-old female who had not attained her menarche and wanted to conceive. She was diagnosed with TS on the basis of clinical and laboratory investigations. The patient was, thereafter, treated for infertility by oocyte donation and conceived successfully.

## Introduction

Turner syndrome (TS) was first reported in the literature in 1938 by Laurel Thatcher Ulrich and Henry Turner [1]. One in 2,500 live-born girls develops TS [2]. It is the most prevalent sex chromosomal defect among women [3]. The second-most prevalent chromosomal abnormality that causes miscarriage is TS [4]. It is a syndrome where one X chromosome is absent completely or partially and is marked in females by a combination of morphological traits and cytogenetic defects [5]. Common symptoms include gonadal dysplasia, learning difficulties, short stature, webbed neck, peripheral edema, lymphedema, renal and cardiovascular defects, and sexual infantilism. Most TS patients experience rapid follicular atresia, which puts them at risk for primary amenorrhea, early ovarian failure, and infertility later in life. Here, we present a rare case of successful pregnancy in a patient with mosaic TS by oocyte donation.

## Case presentation

**Figure 1:**
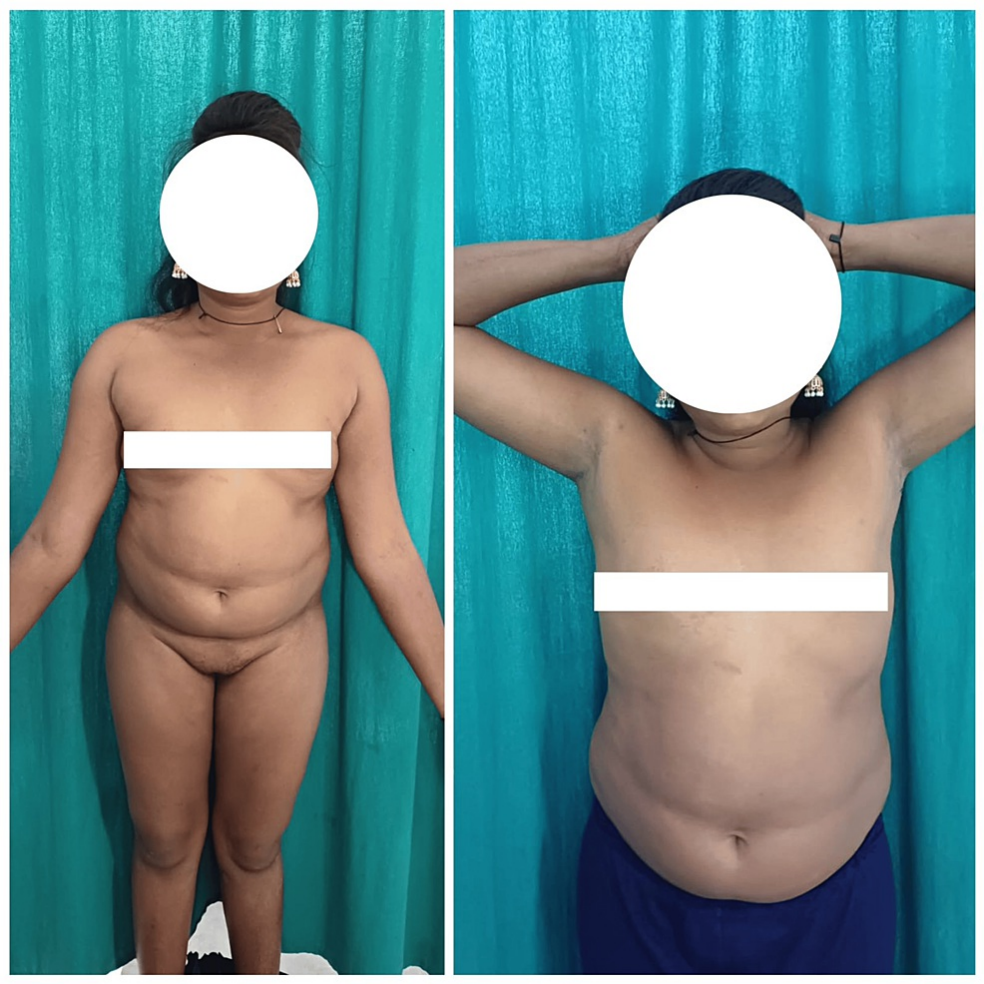
Clinical presentation of Turner syndrome

A 27-year-old female, married for two years, presented to the obstetrics and gynecology outpatient department of our hospital with primary infertility. Upon taking her menstrual history, she revealed that she had never attained menarche. The patient gives a history of consulting a local practitioner for primary amenorrhea in the past; a progesterone challenge was prescribed, which failed to attain menses. Due to a lack of proper knowledge about the condition, she failed to consult the concerned medical fraternity and was not diagnosed. On physical examination, the patient was found to have a height of 140 cm. Other physical characteristics such as a barrel-shaped chest, webbed neck, and widely spaced nipples were present (Figure 1).

The patient was in the pre-pubertal stage of breast and pubic hair growth. On general examination, the patient was vitally stable. There were no signs of pallor, icterus, clubbing, cyanosis, koilonychia, edema, or lymphadenopathy. The systemic examination of the patient is unremarkable. The abdomen was found to be soft, non-tender, and without any signs of organomegaly or palpable masses. On local examination, a ruptured hymen was present, and a vaginal canal was seen. On a bimanual examination, the uterus was found to be small and mobile. Then, she was advised of the mentioned investigations below to rule out the cause. Investigations of the patient are mentioned in Table 1.

**Table 1:** Laboratory parameters in the patient Hb - Hemoglobin, TLC - Total leukocyte count, APTT - Activated partial thromboplastin clotting time, PT - Prothrombin time, INR - International normalized ratio, RBS - Random blood sugar, LFT - Liver function test, KFT - Kidney function test, TSH - Thyroid-stimulating hormone, FSH - Follicle-stimulating hormone, LH - Luteinizing hormone

PARAMETER	VALUE
Hb	11.3 gm%
TLC	9100/mm^3^
Platelet	3 lakhs
APTT	28.3 sec
PT	10.8
INR	0.94
RBS	86 mg/dl
LFT and KFT	WNL
T3	96 ng/dl
T4	6.7 mcg/dl
TSH	2.6 mIU/L
FSH	18.2 mIU/L
LH	1.5 mIU/L
Testosterone	0.16 ng/dl
Ultrasound	Uterus appeared hypoplastic and both ovaries appeared streaked.

**Figure 2:**
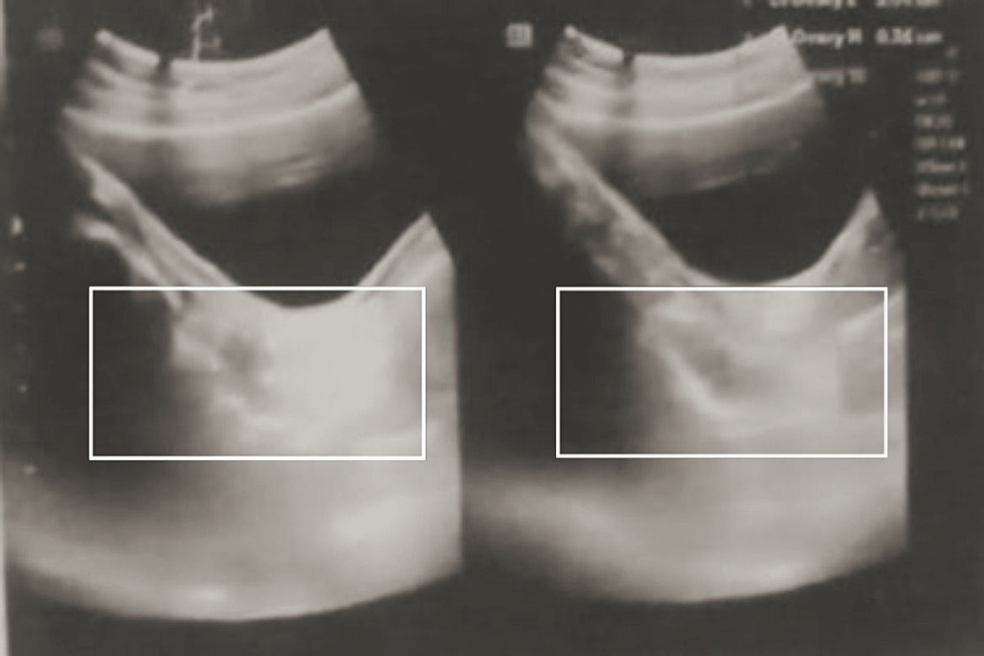
Ultrasonography showing the hypoplastic uterus

**Figure 3:**
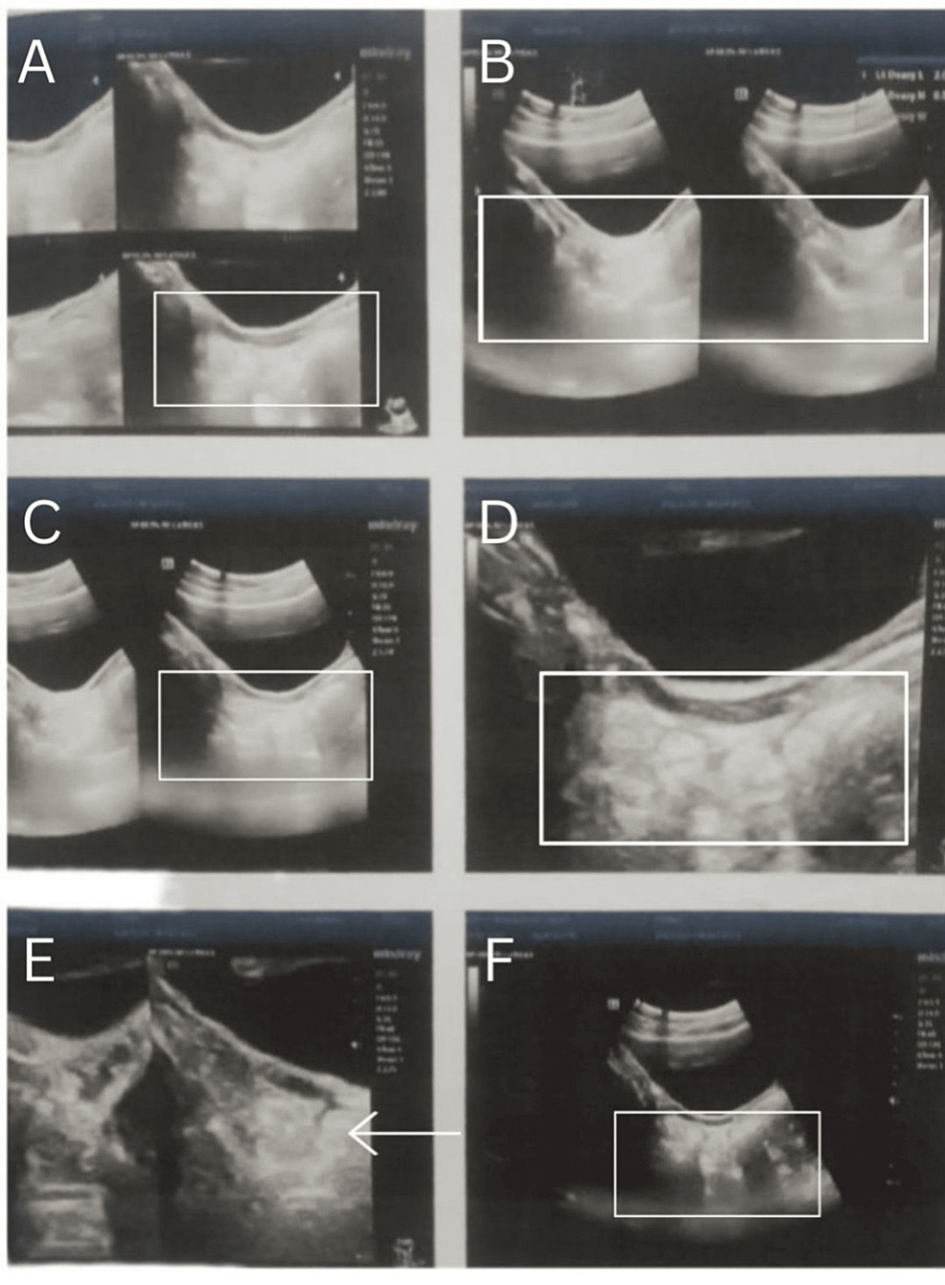
Ultrasonography report of the patient showing the hypoplastic uterus and streak ovaries A, B, and C panels depict the hypoplastic uterus, and D, E, and F panels show the streak ovaries.

Significant lab parameters were found to be high FSH (18.2 mIU/mL) and high LH (2.6 mIU/mL), with normal testosterone of 0.14 ng/dL. Ultrasonographical examination revealed a small, hypoplastic, and anteverted uterus, with longitudinal, anteroposterior, and transverse dimensions of 4.2 x 7.0 x 0.6 cm and an endometrial echo in the midline. Both ovaries had a streak appearance, with dimensions of the right ovary of 1.8 x 0.5 cm and the left ovary of 1.6 x 0.5 cm (Figures 2-3).

**Figure 4:**
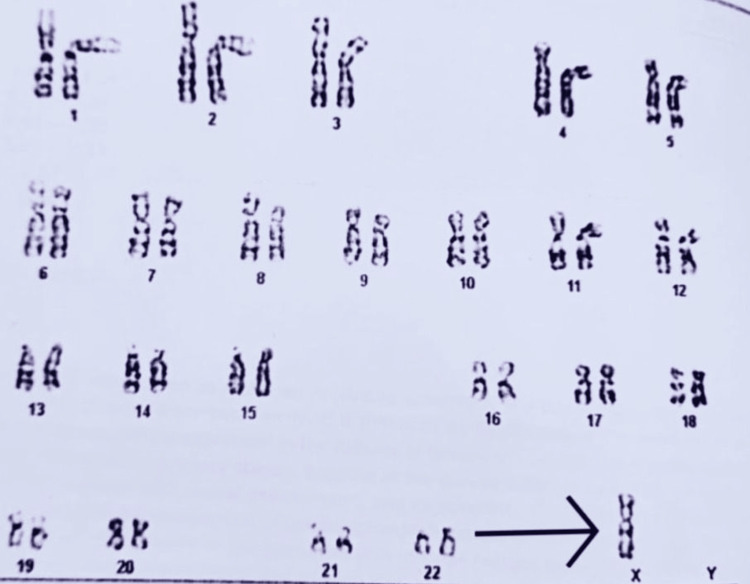
Karyotyping report of the patient

The presence of a hypoplastic uterus and streak ovaries on USG was suggestive of Turner syndrome, which was confirmed by karyotyping of the patient that showed 45, XO (Figure 4).

**Figure 5:**
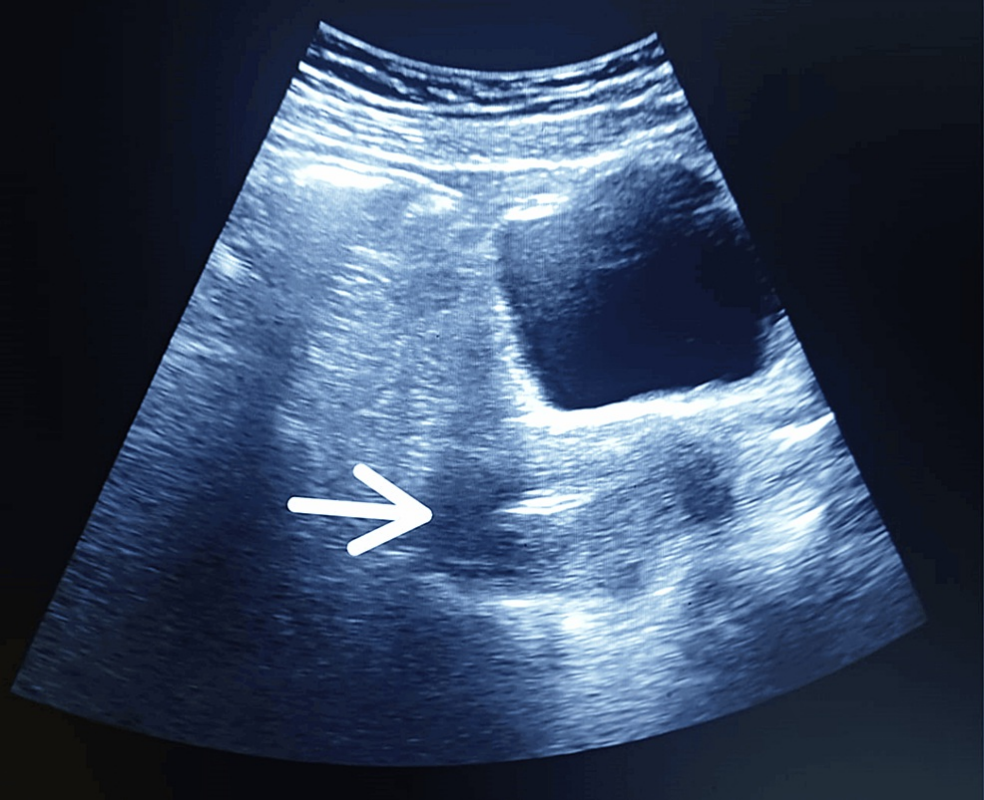
Ultrasonographic depiction of embryo transfer

The patient was then subjected to a 2D ECHO, renal scan, and renal Doppler to rule out other cardiac and renal anomalies that were normal. The patient was counseled about available artificial reproductive technology (ART) options for conceiving, and she opted for oocyte donation. A semen analysis of her husband was carried out and found to be normal. The patient underwent hormonal replacement therapy for three years. As the patient had a hypoplastic uterus, three cycles of estrogen (2 mg BD for 28 days) and progesterone (10 mg OD for the last 10 days) were given. The embryo was prepared using donor oocytes and the husband's sperm in the embryology lab by intracytoplasmic sperm injection. The embryo was formed successfully and transferred on the fourth day of progesterone therapy. The embryo was implanted successfully, as presented by the USG investigation (Figure 5).

Beta-HCG levels were checked on day 14 after embryo transfer, which was 27 mIU/mL, indicating a positive implantation result. Unfortunately, the patient had per vaginal bleeding on day 18 post-embryo transplant, which resulted in an abortion.

## Discussion

TS in females has characteristic features of delayed puberty presenting as primary amenorrhea, short height, shield chest, widely spaced nipples, webbed neck, and typical cytogenetic abnormality (karyotype, 45XO), which was also depicted in this patient. She was eager to conceive and opted for oocyte donation for conception. Pregnancy options spontaneous and ART-based in TS females and their outcomes from the available research literature are listed in Table 2.

**Table 2:** Research reports of pregnant females with Turner syndrome and its outcomes

Author	Article type/Publication year	Results
Topdaği [6]	Case report 2021	Turner syndrome patient with a history of unplanned pregnancies and live births was the subject of discussion.
Yousif et al. [7]	Case Report 2020	Reported an instance of a patient with Turner syndrome who conceived spontaneously.
Sabzervar et al. [8]	Case Report 2020	Reported a case of mosaic Turner who had four spontaneous pregnancies that resulted in three infant deaths/abortions and one healthy child.
Church et al. [9]	Case Report 2014	A case of Turner syndrome with pre-existing insulin-dependent diabetes, hypertension, and primary hypoparathyroidism, despite all these complexities the patient delivered a healthy baby to term.
Kable et al. [10]	Case report 1981	Reported a second pregnancy in a woman with Turner mosaicism and went through the data required for effective counseling.

TS was initially reported 60 years ago and affects approximately 3% of total females who conceive and also being the most prevalent sex chromosomal anomaly in females. A total of 20% of all spontaneously aborted fetuses are reported to have TS [11-13]. Distinctive physical characteristics of the fetus and chromosomal analysis of cells in amniotic fluid can be helpful in the early detection of TS [14]. One-third of the girls are diagnosed with TS due to their small stature in mid-childhood. One or more X chromosomes or their parts are found missing in females affected by TS; occasionally, some of the body sections may have healthy cells having both X chromosomes (46, XX), whereas it can be absent in the rest of the cells (45, X, also known as mosaicism). Though, delayed growth at birth is a commonly observed feature in TS, other specific developmental deficits and their associations are yet to be determined [15]. Classic TS in females is marked by short stature, late or no menarche, thyroid dysfunction, hypoplastic uterus, and bilateral streak ovaries. Skeletal abnormalities and kidney malformations have also been noticed in some cases. Most females are diagnosed as having TS during adolescence or adulthood due to amenorrhea during puberty. Follicular atresia leads to infertility and gonadal insufficiency, although approximately 16% of patients may experience spontaneous puberty [16], including normal ovulatory cycles and a conceiving potential. Premature ovarian failure typically develops in early adulthood [17]. Females having TS have options to conceive attributed to the recent advancements in ART [18]. Numerous studies have shown that patients with TS with mosaicism had higher rates of spontaneous pubertal development and menarche as well as unassisted conception than patients with monosomy X. This syndrome has no known treatments, but symptomatic treatment options are available to alleviate clinical conditions, which include specialized care for related health conditions, such as blood pressure management, thyroid issues, hormonal support, radiological screenings, etc. Physicians should assess gonadotropin levels before initiating estrogen therapy in young females as a supportive treatment.

## Conclusions

Preconception counseling, including cardiac and renal assessment, plays a pivotal role in managing fertility issues in females with TS. Such patients are encouraged to seek psychological or psychiatric assistance to aid social integration, self-sustainment, and achieving contentment with their physical appearance. Patients with TS should be openly counseled on all hazards and challenges that can arise when pregnant and after childbirth with a detailed explanation of all possible fertility preservation strategies and available ART options for the future. Fertility and childbearing are risky for females with TS, and a multidisciplinary management approach is required in order to minimize complications.
